# More Proximal, More Willing to Purchase: The Mechanism for Variability in Consumers’ Purchase Intention Toward Sincere vs. Exciting Brands

**DOI:** 10.3389/fpsyg.2020.01258

**Published:** 2020-06-25

**Authors:** Tingyun Hu, Bing Shi

**Affiliations:** Department of Psychology, Sun Yat-sen University, Guangzhou, China

**Keywords:** brand personality, purchase intention, psychological distance, attachment anxiety, attachment avoidance

## Abstract

Sincerity and excitement are core brand personality dimensions, which capture the majority of consumers’ personality perceptions associated with brands. Previous research has demonstrated that consumers are more willing to purchase sincere brands than exciting brands. The present research addresses the mechanism underlying this variability. A total of four studies were conducted. Study 1 adopted survey-based design and manipulated brand personality by two versions of fictitious coffee brands. Results showed that sincere (vs. exciting) brands elicited more proximal psychological distance and in turn led to higher purchase intention. With a similar procedure, a different sample, and a different product (vacuum cup), Study 2 replicated the pattern demonstrated in Study 1. Moreover, the impact of brand personality upon psychological distance was found to be more prominent among consumers with high (vs. low) levels of attachment anxiety. Because psychological distance is proposed and proved to be a critical variable for mediating the variability in purchase intention, we adopted cognitive computerized tasks in Studies 3–4 to test whether stimuli perceived as sincere and exciting will induce different responses relevant to the perceptions of psychological distance. Study 3 adopted a picture-word version of Stroop task to test whether the automatic activation of personality-priming words would carry various perceptions of psychological distance, and results showed that participants classified distance faster when a close (vs. far) spatial distance matched sincere words and when a far (vs. close) spatial distance matched exciting words. Study 4 adopted the interference task to examine whether visual attention would be affected by personality-priming images, and results indicated a stronger cueing effect and an articulated interference effect for sincere (vs. exciting) figures. This research advances the literature of brand personality by probing the important role of psychological distance and further elaborating on the variability of consumer behavior toward sincere and exciting brands.

## Introduction

Consumers’ perceptions of associating the personality traits with a product/brand and corresponding responses have been documented and examined in the consumer literature (Aaker, 1997; Fournier, 1998). Such symbolic associations that consumers develop and maintain through interactions with products/brands (Chen et al., 2015) are termed as brand personality. Brand personality represents “the set of human characteristics associated with a brand” (Aaker, 1997, p. 347). By personifying brands in this manner, consumers help create and sustain an intimate relationship with the brand (Fournier, 1994; Aaker, 1997). Considering this, marketing practitioners view brand personality as a strategic tool for brand differentiation (Park and John, 2012; Ha and Janda, 2014; Hultman et al., 2015) and a central driver of consumer preference (Guevremont and Grohmann, 2013; Gordan et al., 2016).

Similar to human personality, brand personality is composed of various dimensions (Aaker, 1997; Geuens et al., 2009). Of the five brand personality dimensions developed by Aaker (1997), which represents the most prominent operationalization of brand personality (Eisend and Stokburger-Sauer, 2013; Matzler et al., 2016), sincerity and excitement are considered as the two primary brand personality dimensions, because these two appear to capture the majority of the variance in personality ratings of brands (Aaker, 1997; Aaker et al., 2001), and they also constitute two of the three partner ideals in intimate personal relationships: warmth–trustworthiness and vitality–attractiveness, respectively (Fletcher et al., 1999; Aaker et al., 2004). Accumulating evidence has indicated that consumers are more willing to purchase sincere brands than exciting brands (see a review in Eisend and Stokburger-Sauer, 2013). However, the mechanism underlying this discrepancy across sincere and exciting brands has not been adequately investigated. Considering the prevalence of brand personality perceptions among consumers and the popularity of brand personality promotion strategy in marketing/advertising practices, it is critical to investigate this issue. We aim to address this gap in the present research.

Sincere brands are perceived as warm and down-to-earth (Buss, 1991; Robins et al., 2000; Aaker et al., 2004), whereas exciting brands as energetic and possessing vitality (Aaker et al., 2004). Sincere and exciting brand personalities primarily tap into agreeableness and extraversion of the “big five” human personality traits, respectively (Aaker, 1997). Social psychologists reveal that people are more likely to include individuals with high agreeableness (vs. extraversion) in their inner social circles (Wortman and Wood, 2011). Research in impression formation processes of individuals and groups has demonstrated that people attach more value to sincerity than to excitement (Leach et al., 2007; Brambilla et al., 2013). Moreover, it is indicated that perception of sincerity promotes relationship intimacy (Reis and Patrick, 1996). In marketing landscape, it was suggested that consumers’ relationship with brands is stronger when the brands are perceived to be more sincere (Arya et al., 2019). Hence, sincere (vs. exciting) brands should be perceived as closer or more proximal by consumers.

A number of empirical studies have demonstrated that psychological distance of an object or a person from a perceiver is a key factor in determining his/her judgments of this object or this person (e.g., Harwood and Lin, 2000; Etang et al., 2011; Liberman and Trope, 2014; Thomsen et al., 2016). For instance, participants were more willing to give out money to people who were relatively more proximal toward them (Etang et al., 2011). Psychological closeness/distance constitutes a geographic metaphor used to describe personal experience, involving the abundance/absence of communication with one another (Kreilkamp, 1984). Within the marketing context, psychological distance between consumers and brands suggested the degree of connection or psychological bonds (Story and Hess, 2006; Trope et al., 2007). Recent studies revealed that manipulation of a proximal psychological distance could improve brand trustworthiness and online purchase behaviors, especially for the first purchase encounter with a previously unknown retailer (e.g., Edwards et al., 2009; Lii et al., 2013; Darke et al., 2016). These findings present evidence for the proposition that proximal psychological distance between consumers and a brand should promote consumers’ positive evaluations or purchase intention toward the brand. Taken together, we hypothesize the following:

H_1_: Consumers’ more willingness to purchase sincere (vs. exciting) brands is mediated by the more proximal psychological distance induced by sincere (vs. exciting) brands.

Furthermore, how consumers form relationship with brands should differ by individual characteristics. Considering empirical studies in social psychology indicating that interpersonal relationships are affected by individuals’ attachment styles (e.g., Rom and Mikulincer, 2003; Wei et al., 2005; Levi-Belz and Lev-Ari, 2019), we adopt the attachment theory (Bowlby, 1980) to further elaborating on individual differences in the influence processes underlying consumers’ purchases toward sincere vs. exciting brands.

In the attachment theory, two dimensions (i.e., attachment anxiety and attachment avoidance) are proposed based on individual’s view of self and others, which are likely to determine the types of relationships he/she wants to engage in Bartholomew and Horowitz (1991); Collins and Stephen (1994), Pierce and John (1998) and Bartz and Lydon (2004). Individuals with high attachment anxiety are perpetually preoccupied with their self-worth and self-esteem concerns and direct excessive attention toward attachment figures by using a defensive strategy known as hyperactivation (Mikulincer et al., 2003). Hyperactivation implies greater vigilance of relationship-related behaviors and information. Individuals with high levels of attachment anxiety rely more on external sources to enhance self-worth (Mikulincer et al., 2003) and persist in seeking external comfort, reassurance, and support (Birnbaum et al., 2006). Given that sincerity (vs. excitement) meets more characteristics of an ideal partner for interpersonal relationships (Fletcher et al., 1999; Aaker et al., 2004), sincere brands should be perceived as more comforting and reliable by consumers with high attachment anxiety than by consumers with low attachment anxiety. Therefore, it can be predicted that psychological distance between sincere brands and consumers should be more proximal for individuals with high attachment anxiety than for individuals with low attachment anxiety. Considering the foregoing discussion on the mediation effect of psychological distance on consumers’ purchase preference of sincere over exciting brands, we hypothesize the following:

H_2_: Purchase preference of sincere over exciting brands is more prominent for consumers with high (vs. low) attachment anxiety, which occurs via the more proximal psychological distance of sincere brands toward the consumers.

Regarding attachment avoidance, individuals with high attachment avoidance are characterized by a high degree of self-reliance and desire for autonomy (Mikulincer et al., 2003). Avoidant individuals are reluctant to rely on others. Findings show that individuals with high attachment avoidance tend to have relationships characterized by low levels of emotional involvement and satisfaction (Hazan and Phillip, 1987; Collins and Stephen, 1994; Kirkpatrick and Davis, 1994). In contrast, individuals with low avoidance have a favorable view of others and are interested in pursuing intimate and close relationships (Hazan and Phillip, 1987). Therefore, it is likely a sincerity (vs. excitement) brand personality is perceived as closer toward these consumers given the more consistency with low avoidant consumers’ expectations of brand partners. As thus, we predict the following:

H_3_: Purchase preference of sincere over exciting brands is more prominent for consumers with low (vs. high) attachment avoidance, which occurs via the more proximal psychological distance of sincere brands toward the consumers.

With four studies, we examined whether the more proximal distance perceived by consumers of sincere (vs. exciting) brand personality induces a higher level of purchase intention toward sincere (vs. exciting) brands (H_1_) and, furthermore, whether the impact of brand personality on psychological distance varies across different levels of consumers’ attachment anxiety (H_2_), as well as attachment avoidance (H_3_). Study 1 adopted survey-based design to test H_1_. With a similar procedure and a different sample, Study 2 examined generalizability of the effect of brand personality demonstrated in Study 1 in another product category (vacuum cup) and meanwhile investigated the moderating role of consumers’ attachment style in the impact of brand personality on psychological distance. Specifically, we investigated whether sincere (vs. exciting) brand personality is likely to be more proximal and more appealing to those with higher attachment anxiety or lower attachment avoidance.

Because psychological distance is proposed to be a critical variable for mediating the variability in purchase intention, we adopted cognitive computerized tasks in Studies 3 and 4 to test whether stimuli perceived as sincere and exciting will induce different responses relevant to the perceptions of psychological distance. Based on the assumption that people can automatically process and assess psychological distance of any target (Bar-Anan et al., 2006, 2007), Study 3 adopted a revised Stroop task to test whether the automatic activation of personality-priming words carry various perception of psychological distance. Study 4 adopted the interference task (Shen et al., 2016) to examine whether visual attention would be affected by personality-priming images.

## Study 1

The core object of Study 1 was to test the basic prediction within the marketing context that sincere brands will induce a more proximal psychological distance than exciting brands, and psychological distance mediates the relationship between types of brand personality and purchase intention.

### Materials and Methods

#### Stimuli Selection

We first designed two versions of a website for manipulating the brand personality of a fictitious coffee brand named CARLO. Note that we used a fictitious brand in this research for two reasons: (1) it allowed us to cleanly manipulate the brand personality, while controlling for the brand name across conditions; (2) it enabled us to test if the effect of brand personality on psychological distance and purchase intention was robust enough to emerge in the context of relatively unknown or new brands. We followed Aaker et al. (2004) detailed procedure to manipulate brand personality. Specifically, we varied four key elements of the website: color (soft brown vs. bright red), visuals (sitting dog vs. jumping dog), font (Comic Sans vs. Algerian), and content (family picnic vs. parachuting). Please see Supplementary Appendix A for stimuli.

A pretest (*n* = 46) was conducted to evaluate the effectiveness of the manipulation of the brand personality using a validated scale for brand personality (Aaker et al., 2004). Participants were asked to rate the degree to which each brand was perceived as associated with five measurement items for sincerity traits (“sincere,” “honest,” “wholesome,” “down-to-earth,” “family oriented”; 1 = not at all, 7 = to a great extent; α = 0.85) and five measurement items for exciting traits (“exciting,” “unique,” “young,” “imaginative,” “daring”; α = 0.81). Findings confirmed that participants presented with the sincere website rated CARLO as more sincere (mean = 4.74, *SD* = 0.81) than those presented with the exciting website (mean = 3.66, *SD* = 0.57), *F*(1, 44) = 27.39, *p* < 0.001, η*_*p*_*^2^ = 0.38. Similarly, participants presented with the exciting website rated CARLO as more exciting (mean = 4.72, *SD* = 0.72) than those presented with the sincere website (mean = 3.68, *SD* = 0.64), *F*(1, 44) = 26.35, *p* < 0.001, η*_*p*_*^2^ = 0.38. No significant group difference in ratings of other three brand personality dimensions (competence, sophistication, and ruggedness; Aaker, 1997) for CARLO were detected, *P*’s > 0.10.

#### Participants and Procedure

Ninety-five undergraduate students (37 males, 58 females, mean_age_ = 21.76 years, SD_age_ = 2.49) were recruited to participate in this study. Participants were randomly assigned to view a website page introducing a sincere or exciting brand. The website page for introducing CARLO appeared on a tablet in order to mimic our product search process when shopping online. They were informed with the following scenario: a new coffee product of this brand was being introduced by the company recently, and this study was commissioned on behalf of the company to gain feedback about the product. Participants were further asked to answer survey questions relevant to the presented product. In specific, purchase intention was captured on a two-item scale (“likely to purchase,” “probable to purchase”; 1 = not at all, 7 = to a great extent; α = 0.81). Next, participants reported their perceived psychological distance of the brand from themselves on a three-item, seven-point scale adapted from previous research by Aron et al. (1992) and Liviatan et al. (2006) (“closely associated with the brand,” “similar to users of the brand,” “overlap of self and the brand”; α = 0.93). To control for the possible effect of the product familiarity and health concern on brand preference, product familiarity was measured on a two-item, seven-point scale (“frequently purchase coffee,” “experienced in purchasing coffee”; α = 0.82), and health concern was measured on a single item (“I think coffee is harmful to health”).

It should be noted that, as researchers suggested (Aaker et al., 2004; Sundar and Noseworthy, 2016), consumers see exciting brands as unorthodox and unpredictable and sincere brands as consistent and authentic. It can be predicted that consumers will be aroused more when seeing exciting brands than sincere brands. In order to test whether arousal may be a potential facilitator for purchase discrepancy across sincere and exciting brands, we also included the measurement of arousal here. Arousal was measured on a three-item, seven-point scale adapted from the previous research by Kaltcheva and Weitz (2006) (“This brand attracts my attention,” “This brand arouses me,” “This brand makes me feel exciting”; α = 0.90). We also collected a manipulation check for brand personality (discussed in the pretest). The instrument concluded with basic demographic information.

Upon completion, participants received financial rewards for their time. All of them provided written informed consent to participate in this study.

### Results

#### Manipulation Check

Analyses of sincerity and excitement as a function of brand personality confirmed the effectiveness of the manipulation of brand personality, such that participants perceived the sincere brand to be more sincere (mean = 5.20, *SD* = 0.81) than the exciting brand (mean = 3.56, *SD* = 1.10), *F*(1, 93) = 69.00, *p* < 0.001, η*_*p*_*^2^ = 0.43. Similarly, participants perceived the exciting brand to be more exciting (mean = 5.00, *SD* = 1.00) than the sincere brand (mean = 3.64, *SD* = 0.87), *F*(1, 93) = 50.12, *p* < 0.001, η*_*p*_*^2^ = 0.35.

#### Hypothesis Testing

To explore whether psychological distance mediated the impact of brand personality on purchase intention, we ran mediation analyses following the procedures developed by Baron and Kenny (1986) (Table 1). First, we ran a regression analysis with purchase intention as the dependent variable, brand personality (1 = sincere; 0 = exciting) as the predictor, and product familiarity and health concern as covariates. Results revealed a significant main effect of brand personality (β = 0.39, *t* = 3.93, *p* < 0.001), indicating that participants were more willing to purchase a sincere brand (mean = 4.76, *SD* = 1.24) than an exciting brand (mean = 3.72, *SD* = 1.43).

**TABLE 1:** Results of the mediation analysis in Study 1.

	**β**	***T***	***P***
**Step 1 (DV = purchase intention)**			
Brand personality	0.385	3.926	<0.001
**Step 2 (DV = psychological distance)**			
Brand personality	0.242	2.424	0.01
**Step 3 (DV = purchase intention)**			
Brand personality	0.247	2.982	<0.01
Psychological distance	0.571	6.773	<0.001

*Brand personality (1 = sincerity; 0 = excitement); DV = dependent variable. Coefficients for the control variables including health concern and product familiarity are not presented in the table.*

Second, an analysis of psychological distance as a function of brand personality (1 = sincere; 0 = exciting), with covariates controlled, revealed a significant main effect of brand personality (β = 0.24, *t* = 2.42, *p* = 0.01), indicating that sincere brands were perceived as more proximal (mean = 3.16, *SD* = 1.58) than exciting brands (mean = 2.62, *SD* = 1.42).

When both brand personality and psychological distance were included in the model, the main effect of brand personality became weaker (β = 0.25, *t* = 2.98, *p* < 0.01), as compared to the effect of brand personality in the first model mentioned above (β = 0.39, *t* = 3.93, *p* < 0.001), whereas the effect of psychological distance was significant (β = 0.57, *t* = 6.77, *p* < 0.001). The results of the bootstrapped analysis (Model 4; Hayes, 2012) revealed a significant indirect effect of psychological distance [IE = 0.39, 95% confidence interval (CI) = 0.09–0.72, excluded zero]. These findings are consistent with H_1_, which stated that psychological distance mediates the impact of brand personality (sincerity vs. excitement) on consumers’ purchase intention.

We also tested whether arousal would be a potential mediator for the impact of brand personality. A regression analysis of arousal as the function of brand personality, with covariates controlled, revealed a marginally significant main effect of brand personality (β = −0.18, *t* = −1.73, *p* = 0.09), which indicated that the exciting brand tended to be perceived as more arousal (mean = 3.89, *SD* = 1.50) than the sincere brand (mean = 3.35, *SD* = 1.26). However, we conducted the similar bootstrapped mediation analysis (Model 4; Hayes, 2012) with arousal entered as the mediator, brand personality as the independent variable, and purchase intention as the dependent variable. Results showed that arousal did not mediate the relationship between types of brand personality and consumers’ purchase intention (IE = −0.33, 95% CI = −0.73–0.06, included zero), which indicated that although exciting brands are perceived to be more arousal than sincere brands, arousal does not explain the variability in consumers’ purchase intention toward these two types of brand personality.

### Discussion

Consistent with H_1_, findings in Study 1 showed that psychological distance mediates the impact of brand personality (sincerity vs. exciting) on consumers’ purchase intention. Specifically, compared to exciting, sincere brand personality is perceived as more proximal and induces higher purchase intention. Moreover, arousal was not a significant mediator to underlie the variability in consumers’ purchase intention toward sincere vs. exciting brands. In Study 2, we adopted a different product type (vacuum cup), and we introduced the measurement of individuals’ attachment anxiety and avoidance, in order to test H_2_–H_3_.

## Study 2

The core objectives of Study 2 were (1) to replicate the key findings of Study 1 with another product category and (2) to investigate the moderating role of consumers’ attachment style in the impact of brand personality on purchase intention and test the mediating role of psychological distance. Specifically, we hypothesized that, under the condition of high anxiety or low avoidance, individuals perceive sincere brand as more proximal and exhibit much more preference for sincere (vs. exciting) brands.

### Materials and Methods

#### Stimuli Selection

Two versions of an advertisement featuring a vacuum cup launched by a fictitious brand were created to manipulate the brand personality. Similar to Study 1, brand personality was manipulated via color, font, and other brand elements. In addition, the tagline in the sincere condition was “KARLO creates a healthy life with you,” whereas in the exciting condition it was “Enjoy a vital life with KARLO!” Please see Supplementary Appendix B for stimuli.

Similar to Study 1, a pretest (*n* = 41) was conducted to confirm the effectiveness of brand personality manipulation. Participants were asked to rate the degree to which each brand was associated with five measurement items for sincerity traits sincerity traits (“sincere,” “honest,” “wholesome,” “down-to-earth,” “family oriented”; 1 = not at all, 7 = to a great extent; α = 0.85) and with five measurement items for exciting traits (“exciting,” “unique,” “young,” “imaginative,” “daring”; α = 0.81). The present confirmed that participants presented with the sincere advertisement rated KARLO as more sincere (mean = 4.90, *SD* = 0.78) than those presented with the exciting website (mean = 4.04, *SD* = 0.48), *F*(1, 39) = 17.80, *p* < 0.001, η*_*p*_*^2^ = 0.31. Similarly, participants presented with the exciting advertisement rated KARLO as more exciting (mean = 4.60, *SD* = 0.81) than those presented with the sincere advertisement (mean = 3.70, *SD* = 0.54), *F*(1, 39) = 17.88, *p* < 0.001, η*_*p*_*^2^ = 0.31. No significant group differences in ratings of other three brand personality dimensions (competence, sophistication, and ruggedness; Aaker, 1997) for KARLO were detected, *P*’s > 0.10.

#### Participants and Procedure

One hundred thirty-five college students (51 males, 84 females, mean_*age*_ = 20.49 years, SD_*age*_ = 2.22) participated and received financial rewards. None of them have participated in Study 1. Study 2 had a brand personality (sincere vs. exciting) by attachment-style (continuous) mixed-subjects design. Similar to Study 1, brand personality was manipulated in the laboratory using the context of a new product launch. Participants were told to imagine that they were selecting a vacuum cup for themselves and would be exposed to an advertisement introducing a new vacuum cup launched recently, which conveyed either a sincere or an exciting brand personality. After seeing the stimuli, participants were asked to complete a questionnaire consisting of the product evaluation similar to Study 1 and measurements of the personality traits. Purchase intention was assessed on a single item (“likely to purchase”; 1 = not at all, 7 = to a great extent). Perceived psychological distance was measured as in Study 1 (α = 0.78). Attachment style was measured on a revised version of The Revised Experiences in Close Relationships measure (ECR-R) developed by Brennan et al. (1998). Participants stated their level of agreement to statements that assessed their attachment anxiety (“I’m afraid that my friends/partner will not want to stay with me,” “I often worry that my friends/partner doesn’t really love me,” “I worry that friends/partner won’t care about me as much as I care about them,” “I really worry about being abandoned”; α = 0.82), as well as their attachment avoidance (“I prefer not to be too close to a friend/partner,” “I hope to be independent,” “I feel anxious when I am being too close to my friend/partner,” “I hope I don’t rely too much on my friends/partner”; α = 0.68). To control for the possible effect of the product familiarity, product familiarity was measured on a 7-point item (“familiar with vacuum cups”). Finally, the instrument concluded with the manipulation check for brand personality mentioned in the pretest and the collection of basic demographic information.

### Results

#### Manipulation Check: Brand Personality

Analyses of sincerity and excitement as a function of brand personality confirmed the manipulation of brand personality, such that participants perceived the sincere brand as more sincere (mean = 5.21, *SD* = 0.77) than the exciting brand (mean = 4.09, *SD* = 1.20), *F*(1, 130) = 39.88, *p* < 0.001, η*_*p*_*^2^ = 0.24. Similarly, participants perceived the exciting brand as more exciting (mean = 4.82, *SD* = 1.16) than the sincere brand (mean = 3.48, *SD* = 0.92), *F*(1, 128) = 53.60, *p* < 0.001, η*_*p*_*^2^ = 0.29.

#### Hypotheses Testing

We propose that purchase preference of sincere over exciting brands is more prominent for consumers with high (vs. low) attachment anxiety, which occurs via the more proximal psychological distance of sincere brands from the consumers (H_2_) and propose a similar pattern for consumers with low (vs. high) attachment avoidance (H_3_). In other words, we proposed a nomological network with a mediated moderation.

Because attachment anxiety and attachment avoidance are continuous variables, we first ran a regression analysis with purchase intention as the dependent variable, and mean-centered attachment anxiety and attachment avoidance, brand personality (abbreviated as BP; 1 = sincere; 0 = exciting), and their interactions (BP × attachment anxiety; BP × attachment avoidance) as predictors. Regression analyses revealed main effects of BP (β = 0.36, *t* = 4.49, *p* < 0.001) and attachment anxiety (β = -0.38, *t* = -3.19, *p* < 0.01). No other main effect was detected. More importantly, a significant two-way interaction of BP × attachment anxiety was identified (β = 0.45, *t* = 3.79, *p* < 0.001). Simple slope analyses confirmed that attachment anxiety predicted increased purchase intention toward the sincere brand (β = 0.28, *t* = 2.34, *p* < 0.05) and decreased purchase intention toward the exciting brand (β = −0.38, *t* = −3.24, *p* < 0.01). However, the two-way interaction of brand personality × attachment avoidance failed to be significant (β = −0.08, *t* = −0.70, *p* = 0.49).

Next, an analysis of psychological distance, as a function of brand personality, mean-centered attachment anxiety and attachment avoidance, and their interaction (BP × attachment anxiety; BP × attachment avoidance), with the covariate controlled, yielded a significant main effect of brand personality (β = 0.23, *t* = 2.83, *p* < 0.01) and a marginally significant main effect of attachment anxiety (β = −0.23, *t* = −1.88, *p* = 0.06). No other significant main effect was detected. More importantly, a significant two-way interaction of brand personality × attachment anxiety was identified (β = 0.41, *t* = 3.42, *p* = 0.001). Simple slope analyses confirmed that attachment anxiety predicted increased psychological proximity toward the sincere brand (β = 0.39, *t* = 3.31, *p* < 0.01) and decreased psychological proximity toward the exciting brand (β = −0.24, *t* = −1.98, *p* = 0.05). However, the two-way interaction of brand personality × ×attachment avoidance failed to be significant (β = −0.11, *t* = −0.89, *p* = 0.32).

To determine whether psychological distance accounted for the moderating effect of attachment anxiety on purchase intention, we constructed a regression model with purchase intention as the dependent variable, and both the interaction of brand personality × attachment anxiety and psychological distance included as predictors. The effect of brand personality became weaker (β = 0.24, *t* = 3.41, *p* < 0.01) as compared to the effect detected in the first model mentioned above (β = 0.36, *t* = 4.49, *p* < 0.001). More importantly, the coefficient for the interaction of BP × attachment anxiety became weaker (β = 0.23, *t* = 2.21, *p* < 0.05) as compared to the effect detected without the mediator included (β = 0.45, *t* = 3.79, *p* < 0.001), whereas the effect of psychological distance was significant (β = 0.52, *t* = 7.03, *p* < 0.001). A bootstrapped mediated moderation analysis (Hayes, 2012; Model 8) revealed the interaction effect of BP × attachment anxiety on purchase intention was indeed mediated by psychological distance (index of mediated moderation = 0.35, 95% CI = 0.18–0.54, excluded zero). Consistent with the preceding information, conditional mediation analyses revealed that psychological distance mediated the positive impact of attachment anxiety on the purchase intention toward the sincere brand (IE = 0.22, 95% CI = 0.11–0.34, excluded zero) and meanwhile mediated the negative impact of attachment anxiety on the purchase intention toward the exciting brand (IE = −0.13, 95% CI = −0.26 to −0.01, excluded zero). These results were consistent with H_2_. Inconsistent with H_3_, a bootstrapped mediated moderation analysis (Hayes, 2012; Model 8) revealed the interaction effect of BP × attachment avoidance on purchase intention was not mediated by psychological distance (IE = −0.12, 95% CI = 0.32–0.07, included zero) (Table 2).

**TABLE 2:** Results of mediated moderation analysis in Study 2.

	**β**	***t***	***P***
**Step 1 (DV = purchase intention)**			
BP	0.364	4.493	<0.001
Attachment anxiety	–0.375	–3.185	<0.01
Attachment avoidance	–0.018	–0.150	0.881
BP × attachment anxiety	0.445	3.785	<0.001
BP × attachment avoidance	–0.083	–0.700	0.416
**Step 2 (DV = psychological distance)**			
BP	0.234	2.829	<0.01
Attachment anxiety	–0.227	–1.883	0.062
Attachment avoidance	0.053	0.437	0.663
BP × attachment anxiety	0.411	3.419	0.001
BP × attachment avoidance	–0.122	–1.000	0.319
**Step 3 (DV = purchase intention)**			
BP	0.241	3.406	<0.01
Attachment anxiety	–0.257	–2.532	<0.05
Attachment avoidance	–0.046	–0.450	0.653
BP × attachment anxiety	0.230	2.208	<0.05
BP × attachment avoidance	–0.020	–0.195	0.846
Psychological distance	0.522	7.026	<0.001

*BP, brand personality (1 = sincerity; 0 = excitement); DV, dependent variable; Coefficients for the control variable of product familiarity are not presented in the table.*

### Discussion

Results of Study 2 not only replicated findings of Study 1 with a different product category, but also supported our H_2_ on the moderating role of consumers’ attachment anxiety. Specifically, the impact of brand personality upon psychological distance is more prominent among consumers with high (vs. low) levels of attachment anxiety, and therefore sincere (vs. exciting) brands are more appealing to consumers with higher level of attachment anxiety. H_3_ was not supported in our study, suggesting that attachment avoidance does not moderate the impact of brand personality on psychological distance or purchase intention. The reason accounting for this result probably lies in that although individuals with high levels of attachment anxiety are afraid of developing intimate relationships, avoidant style individuals do not shun social contact altogether (Bartholomew and Horowitz, 1991; Roisman, 2006). It can be indicated from our study that how avoidant individuals evaluate the self-brand relationship is different from how they evaluate intimate relationships, in a way that avoidant individuals do not transfer the fear of developing intimate relationships to purchasing a psychologically proximal brand.

Within the marketing context, Studies 1 and 2 clarified that the proximal psychological distance induced by sincere (vs. exciting) brands is a critical variable for mediating the variability in consumers’ purchase intention toward sincere and exciting brands. In Studies 3 and 4, we will further use cognitive computerized cognitive tasks to examine the effect of brand personality on psychological distance, an important driver of consumer behaviors.

## Study 3

The purpose of Study 3 is to test the effect of personality-priming words on psychological distance using a cognitive paradigm. Our study tool was a picture-word version of the Stroop task (e.g., Arieh and Algom, 2002; Shaki and Algom, 2002). The Stroop task is a classic measure to test the selectivity of attention (indeed, its failure), to a relevant aspect of the stimuli. In this study, the picture served to create depth and conveyed various lengths of distance perceived by an observer (i.e., participant) of the word from himself/herself. A target Chinese word (i.e., a personality-priming word) appeared in the picture, which was located either near or far away from the observer. Participants were asked to complete a distance classification task with respect to the position of the target word. It is important to emphasize that the personality-priming words themselves were not words directly related to distance in any literal sense. Thus, the personality-priming words and their locations did not form Stroop-like stimuli unless the symbolic meanings of words perceived and instantly processed by the observer are associated with psychological distance.

### Materials and Methods

#### Participants and Design

Nineteen college students (9 males, 10 females, mean_*age*_ = 20.42 years, SD_*age*_ = 2.09) were recruited to participate in this study. All of them were native Chinese speakers and did not have reading disorder, with normal or rectified vision. Study 3 adopted a 2 (brand personality: sincerity vs. excitement) ×2 (location: near vs. far) within-subjects design.

#### Stimuli Selection

Through Google’s images search tool, we selected two images of alleys with rolling hills that conveyed a clear depth perception, so that participants would be able to easily report the spatial location of an object on the picture. We made two versions of each image, one with an arrow that pointed to a relatively distal location and one with an arrow that pointed to a relatively proximal location. The printed word appeared inside the arrow, in black (font = “song” typeface, which is popularly adopted for Chinese words). The font size of the words was 35-point when they were printed on a spatially proximal arrow and 70-point when they were printed on a spatially distal arrow. Words were selected from brand personality scale developed by Aaker (1997) and constituted an initial pool of 8 items representing the two brand personality dimensions.

#### Procedure

Displays were generated by a computer attached to a 24-inch monitor, using 2,560 × 1,440 resolution graphics mode. To reduce head movement, a chin rest was used. The distance between the eyes of the participants and the top/bottom of the monitor was 70 and 74 cm, respectively.

**FIGURE 1:**
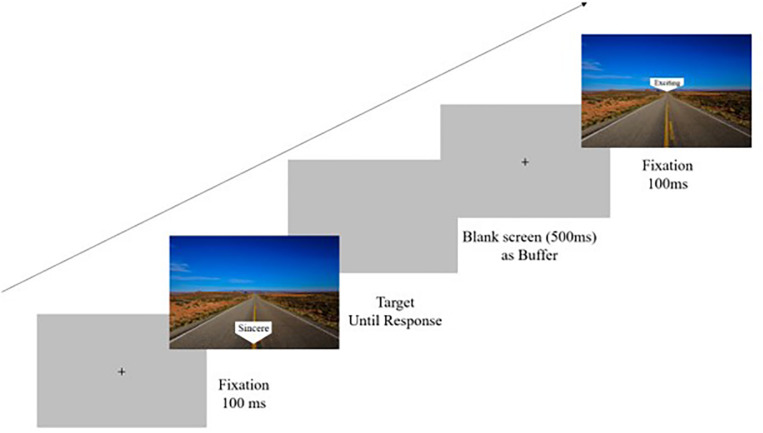
Procedure of experiment 3.

Participants performed the task in individual cubicles. Each participant was first presented with an example – one of the images selected randomly for each participant. They were informed that they would next see similar images with clear depth perspectives and with similar arrows pointing to either a proximal or a distal location in the image. In the experiment trials, participants were requested to respond according to the location of the arrow, and the reaction time of each trial was recorded. Participants’ responses were collected via the computer keyboard. Half of the participants were requested to respond with pressing “S” to indicate proximal spatial location and with pressing “K” to indicate distant spatial location. The response requests were reversed for the other half of the participants. It was made clear to the participants that they would probably have no problem in discriminating between proximal and distal locations, because proximal arrows were always very close to the most proximal location in the image, and distal arrows were always very close to the most distal location in the image. Participants were informed that the words, printed on the arrows, were irrelevant to the current task. The stimuli remained on the screen until the participant responded. The intertrial interval between participant’s press and the display of the next stimulus was 500 ms. Error trials were followed by a 500 ms feedback beep. Thirty-two trials (2 images ×2 locations × 8 words) appeared randomly (Figure 1).

### Results

Reaction times exceeding the mean of all correct responses by more than 3 standard deviations were excluded. Less than 1% of all observations (six trials) were removed. The mean reaction time data were submitted to a 2 (brand personality: sincere vs. exciting) × 2 (locations: near vs. far) within-subjects analysis of variance (ANOVA). The main effect of brand personality was significant, *F*(1, 18) = 6.64, *p* < 0.05, η*_*p*_*^2^ = 0.27, but that of the location was not significant, *F*(1, 18) = 0.04, *p* = 0.84, η*_*p*_*^2^ = 0.002. More importantly, a significant interaction of location and brand personality was detected, *F*(1, 18) = 6.37, *p* < 0.05, η*_*p*_*^2^ = 0.26, which documented the presence of an appreciable Stroop effect in this study. Participants classified distance faster when it matched the brand personality’s perceived psychological distance (for congruent trials including sincere words located more proximal and exciting words farther away: mean = 696.64, *SD* = 205.13) than when they mismatched (for incongruent trials including sincere words located farther away and exciting words more proximal: mean = 746.00, *SD* = 252.67). The Stroop effect accounted to 49.36 ms. Specifically, participants classified the distance faster when sincere words were physically proximal (mean = 674.54, *SD* = 48.69) than when they were far (mean = 727.99, *SD* = 58.29), *F*(1, 18) = 3.73, *p* = 0.07, η*_*p*_*^2^ = 0.17. Conversely, participants classified distance faster when exciting words were physically far (mean = 718.75, *SD* = 46.12) than when they were proximal (mean = 764.99, *SD* = 58.90), *F*(1, 18) = 4.35, *p* = 0.05, η*_*p*_*^2^ = 0.20.

### Discussion

With the use of a revised Stroop task, the results of Study 3 mirrored the effect of brand personality on perceived psychological distance documented in Studies 1 and 2. The results of the picture-word version of Stroop task clearly showed that stimuli featuring sincerity are perceived to be more proximal than those featuring excitement. In the following study, we adopted the interference task to examine the influence of the variability in psychological distance induced by different brand personality on social-based attention.

## Study 4

Study 4 employed an interference task to investigate whether brand personality would modulate visual attention. In each trial of the interference task, a cue letter would be presented on one side of the screen. Then a same letter (in compatible trials) or a different letter (in incompatible trials) was presented on the other side of the screen. Participants were asked to respond according to the second letter presented via pressing the computer keyboard. Typically, participants’ responses to the target are more rapid in compatible than in incompatible trials. Faster response in compatible trials reflects a stronger cueing effect of the consistent information, and slower response in incompatible trials reflects an articulated interference effect of inconsistent information.

Study 4 adopted a 2 (compatibility: compatible vs. incompatible) × 2 (personality: sincerity vs. excitement) within-subjects design. Personality was manipulated by two versions of cartoon characters featuring sincerity and excitement respectively. Studies 1–3 demonstrated that stimuli featuring sincerity would induce a more proximal psychological distance than stimuli featuring excitement. In this case, when stimuli featuring sincerity appear, individuals would be more sensitive to the following consistent information and would be interfered with more by the following inconsistent information. As thus, a stronger cueing effect and an articulated interference effect would be detected in the sincerity personality condition than the excitement personality condition.

### Materials and Methods

#### Participants and Design

Forty-one students (18 males, 23 females, mean_*age*_ = 19.63 years, SD_*age*_ = 1.51) participated in this study. None of them have participated in the studies above. Study 4 adopted a 2 (personality: sincerity vs. excitement) × 2 (compatibility: compatible vs. incompatible) within-subjects design.

#### Stimuli Selection

Two types of cartoon figures were designed to symbolize different kinds of personality. A pretest (*n* = 23) was conducted to confirm the effectiveness of the manipulation. Participants were asked to rate the degree to which each cartoon figure had sincerity traits and exciting traits, respectively (1 = not at all, 7 = to a great extent). Results showed that sincere figures (e.g., a friendly male in suits) were perceived as more sincere (mean = 4.91, *SD* = 1.19) than exciting figures (mean = 3.63, *SD* = 1.02), *F*(1, 22) = 33.86, *p* < 0.001. Conversely, exciting figures (e.g., a cool male in sportswear) were perceived as more exciting (mean = 4.98, *SD* = 1.15) than sincere figures (mean = 3.27, *SD* = 1.16), *F*(1, 22) = 72.86, *p* < 0.001.

#### Procedure

**FIGURE 2:**
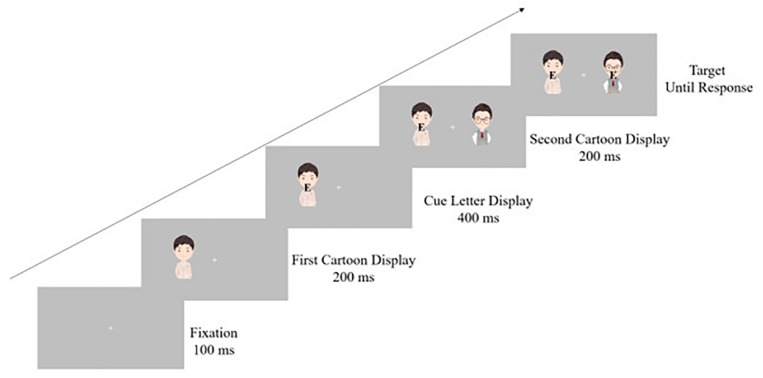
Procedure of experiment 4. This figure explains the procedure for the compatible condition with sincerity-priming features. Participants were asked to make a response as quickly as possible when the target letter appeared.

Participants were tested individually while seated approximately 60 cm from the screen. All displays were presented on a gray background on a 24-inch monitor, using 2,560 × 1,440 resolution graphics mode. To reduce head movement, a chin rest was used. Each trial began with a presentation of a cartoon figure on one side of the screen (either left or right). The fixation was presented at the center of the screen at the same time; the visual angle between the fixation and the cartoon figure was 2°. Being stationary for 200 ms, a letter (*N* or *E*) was then presented on the existing figure. After an interval of 400 ms, another cartoon figure of the same type was presented on the other side of the screen, embedded with the same or a different letter. Participants were asked to report whether the second letter was an *N* or an *E* as quickly as possible by pressing the keyboard. In half the trials, the target and distractor were compatible (i.e., both *N* or both *E*); in the remaining half of the trials, the target and distractor were incompatible. The target and the distractor remained visible until a response was made. Participants were explicitly informed that the cartoon figures displayed were irrelevant of the task and could be ignored. The appearance of the cartoon type was in random sequence (either sincere or exciting). A 2 (compatibility) × 2 (personality) within-subjects design was used. Each participant completed 80 total trials, with 20 trials in each condition. The trials were presented at random (Figure 2).

### Results

A 2 (compatibility) × 2 (personality) repeated-measures ANOVA indicated a significant main effect of compatibility, *F*(1, 40) = 33.48, *p* < 0.001, η*_*p*_*^2^ = 0.46, but no main effect of personality, *F*(1, 40) = 0.39, *p* = 0.54, η*_*p*_*^2^ = 0.01. As expected, the interaction between these two variables was significant, *F*(1, 40) = 10.11, *p* < 0.01, η*_*p*_*^2^ = 0.20. To compare the cueing effect across different types of personality, mean Response time (RT) of the compatible trials were submitted to a paired-samples *t*-test. Results confirmed that participants responded faster in the sincerity personality condition [*t*(40) = -2.39, *p* < 0.05, η*_*p*_*^2^ = 0.13]. To compare the interference effect, a paired-samples *T*-test of mean RT of the incompatible trials revealed that participants responded slower in the sincerity personality condition [*t*(40) = 2.80, *p* < 0.001, η*_*p*_*^2^ = 0.16]. These results indicated a stronger cueing effect, as well as a stronger inference effect in the sincerity (vs. excitement) personality condition.

### Discussion

Results in Study 4 implied that personality-priming images could modulate the social-based visual attention. Specifically, compared to excitement, images featuring sincerity lead to a facilitation of responses with a cueing letter and a delayed response with an interferent letter, consistent with our finding, which stated that sincerity personality is perceived as more proximal than excitement personality.

## General Discussion

Accumulating evidence has shown that consumers are more willing to purchase sincere brands than exciting brands. The present study addresses the mechanism underlying this variability, taking a fresh perspective from psychological distance. Results demonstrate that sincere brands are perceived to be more proximal than exciting brands, such proximal psychological distance inducing more willingness to purchase sincere brands than to purchase exciting ones (Study 1). Study 2 further illustrated that attachment anxiety but not attachment avoidance affects variability in consumers’ purchase intention toward brands of different personality. Specifically, purchase preference of sincere over exciting brands is more salient among consumers with high attachment anxiety as compared to consumers with low attachment anxiety. This pattern is found to occur via the more proximal psychological distance as well.

With adoption of cognitive computerized tasks, in Studies 3 and 4, we confirmed that stimuli perceived as sincere and exciting induce different responses relevant to the perceptions of psychological distance. The picture-word version of the Stroop task was adopted, and the results showed that sincere and exciting words indeed carry underlying meanings associated with psychological proximity and psychological distance, respectively (Study 3). Moreover, compared to stimuli featuring excitement, a stronger cueing effect and an articulated interference effect were detected for stimuli featuring sincerity (Study 4).

Our study makes two major contributions. First, we identify and investigate the mechanism underlying the variability in consumers’ willingness to purchase toward sincere and exciting brands. We prove that psychological distance is a critical variable for mediating the variability in purchase intention and accounting for the interaction effect of attachment style by brand personality on purchase intention. The results of our studies offer valuable insights on brand positioning. Sincere personality traits associated with a specific brand would improve the development of the consumer–brand relationship and consumers’ purchase behaviors, especially for attachment anxiety styles. Second, to our knowledge, this research is among the first few to introduce psychological distance and relevant cognitive paradigms into the brand personality marketing literature. Past studies have shown that spatial closeness has a direct influence on judgments of interpersonal connectedness and emotional attachment (Liberman et al., 2007a, b; Zhang and Wang, 2009). For example, it was indicated that salience of semantic concepts related to physical closeness (e.g., “nearby,” “local”) can lead to a closer social perception toward others (Williams and Bargh, 2008). Indeed, in our daily language, we often use “closest” friend to describe a person who cares about us the most or with whom we have a mutually strong and enduring friendship. Our present study shows that, by associating brands with human personality traits, consumers can interact with brands in ways similar to interpersonal relationship partners and often rely on psychological distance to make their purchase decisions. More importantly, findings of cognitive paradigms demonstrate the effectiveness of psychological distance perception triggered by different symbolism of brand personality, including words and images.

Several areas await future investigations. First, important distinctions should be made between when consumers use a brand as means to signal self-concept and as a relationship partner with which to interact. For example, if an exciting brand is viewed as a means to help express the self (e.g., “I am imaginative”), then consumers may be motivated to “get close to the brand” so that the boundary between the self and the brand is blurred. In contrast, if consumers view brands as relationship partners, they would prefer sincere brands over exciting brands when consumers want to develop stable social relationships. When and how each process occur warrant further study. Second, we have only examined two types of brand personality. Future research could look at the full ranges of personalities that brands may be associated. It would be interesting to investigate whether there is significant difference in psychological distance between sophisticated, competent, and rugged brands and the following influence on consumers’ purchase behaviors.

## Conclusion

With the use of self-report measurements and cognitive paradigms, our research advances the literature of brand personality by probing the important role of psychological distance. Compared to exciting, sincere brands are perceived to more proximal and psychological distance mediating the relationship between brand personality (sincerity vs. excitement) and consumers’ purchase intention. Moreover, the impact of brand personality on psychological distance and purchase intention is more prominent for consumers with higher attachment anxiety. In summary, our research further elaborates on the variability of consumer behavior toward sincere and exciting brands and contributes to the broader discussion about the driving force of psychological distance in consumer behaviors.

## Data Availability Statement

The datasets generated for this study are available on request to the corresponding author.

## Ethics Statement

Ethical review and approval was not required for the study on human participants in accordance with the local legislation and institutional requirements. The patients/participants provided their written informed consent to participate in this study.

## Author Contributions

TH and BS conceived of the idea and designed the studies. TH collected and analyzed all the data. BS mentored the whole processes. TH and BS approved the final manuscript. All authors contributed to the article and approved the submitted version.

## Conflict of Interest

The authors declare that the research was conducted in the absence of any commercial or financial relationships that could be construed as a potential conflict of interest.
