# Supplementary material for: More Proximal, More Willing to Purchase: The Mechanism for Variability in Consumers’ Purchase Intention Toward Sincere vs. Exciting Brands
Source: Front Psychol. 2020 Jun 25;11:1258. doi: 10.3389/fpsyg.2020.01258 (PMC7330119; doi:10.3389/fpsyg.2020.01258)
Supplement: Supplementary file 1 [file Data_Sheet_1.pdf]

Appendix A

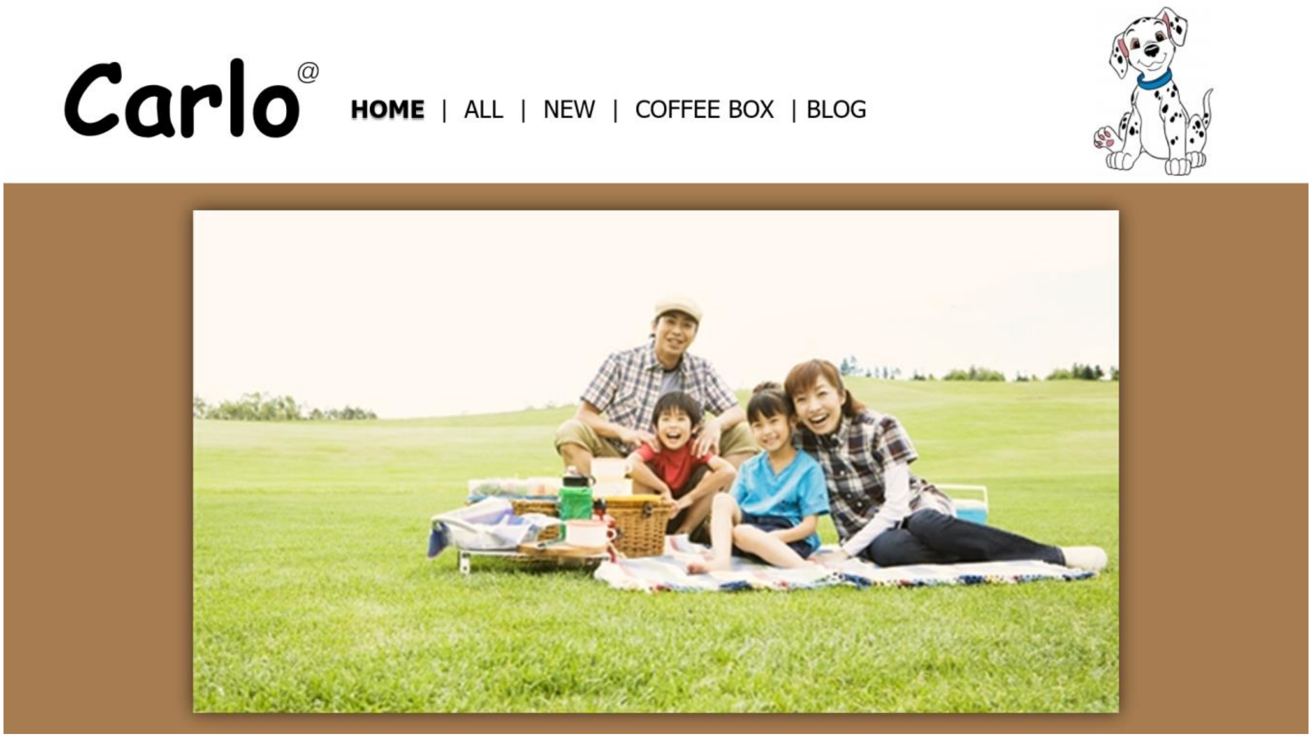

Figure A1 Stimuli for Study 1. Sincere version of Carlo (The website was shown in Chinese for Chinese participants).

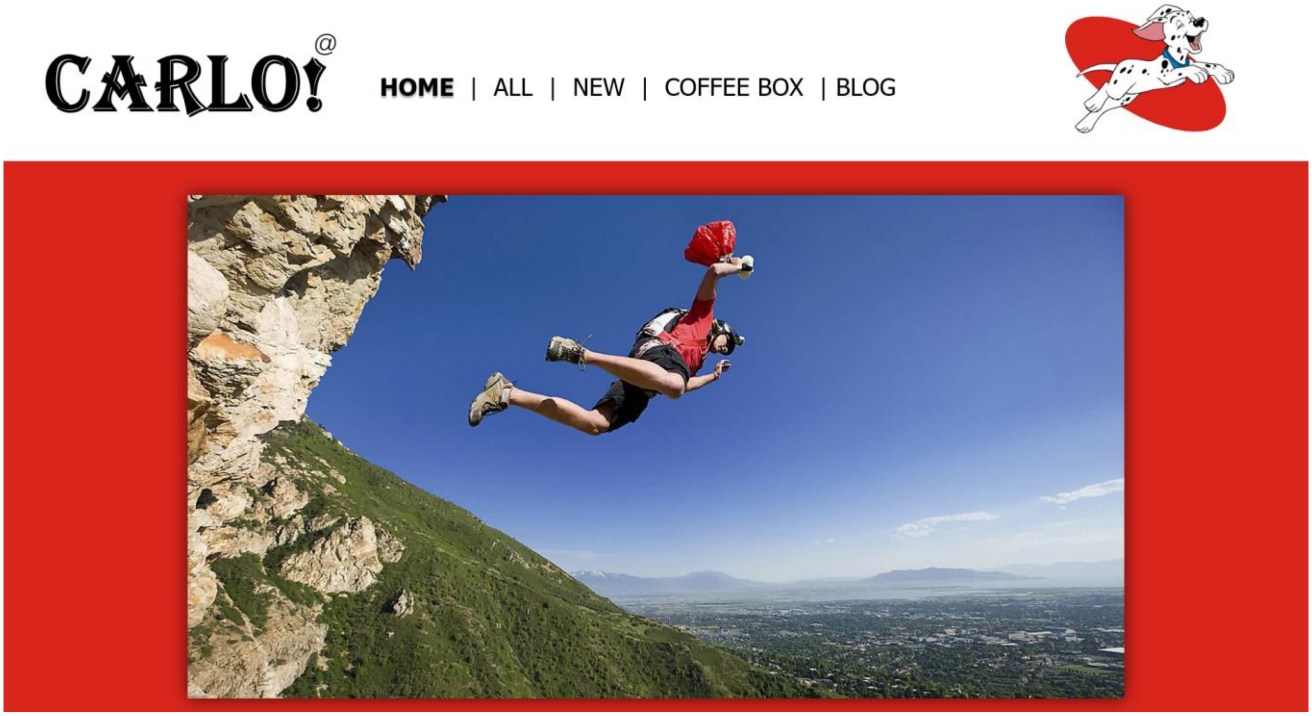

Figure A2 Stimuli for Study 1. Exciting version of Carlo (The website was shown in Chinese for Chinese participants).

Appendix B

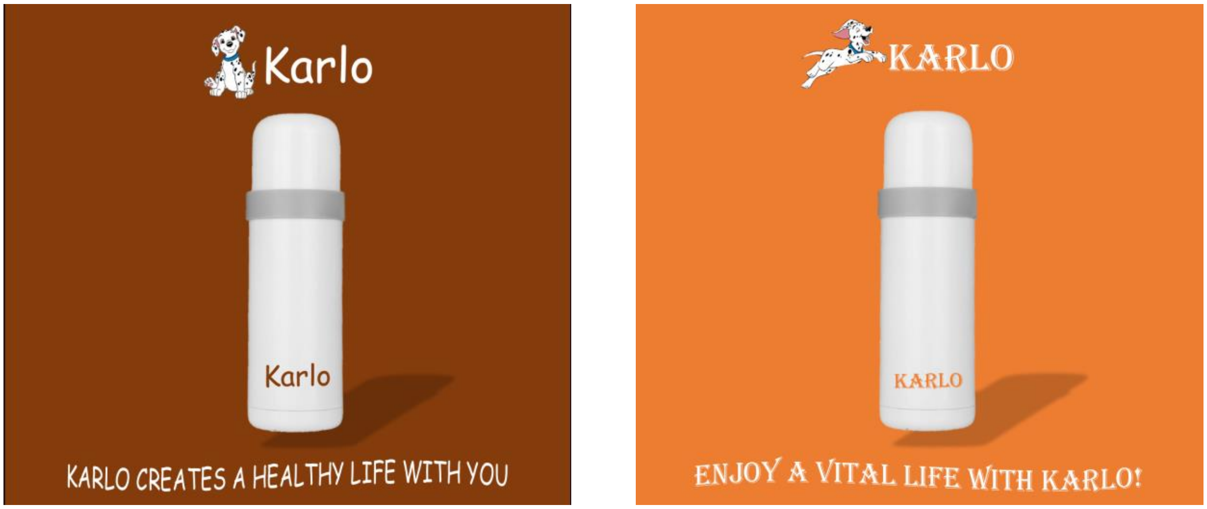

Figure B1 Stimuli for Study 2. Sincere version of Karlo on the left and exciting version of Karlo on the right. (The above advertisements were shown in Chinese for Chinese participants)
